# The direct and indirect effect of neuroticism on work engagement of nurses during COVID-19: A temporal analysis

**DOI:** 10.3389/fpsyg.2022.947887

**Published:** 2022-10-11

**Authors:** Mit Vachhrajani, Sushanta Kumar Mishra, Himanshu Rai, Amit Paliwal

**Affiliations:** ^1^Indian Institute of Management Indore, Indore, India; ^2^Indian Institute of Management Bangalore, Bangalore, Karnataka, India; ^3^Deutsche Gesellschaft für Internationale Zusammenarbeit (GIZ) GmbH, New Delhi, India

**Keywords:** neuroticism, work engagement, beneficiary contact, fear of stigma, nurses, COVID-19

## Abstract

Healthcare professionals such as nurses faced a tough time during the pandemic. Despite the personal and professional challenges, they contributed immensely during the pandemic. However, there were variations in nurses’ work engagement during the pandemic. One reason could be their personality, especially neuroticism. Neuroticism represents individuals’ proneness to distress in stressful situations, such as COVID-19. Hence, understanding how and in which conditions neuroticism influences work engagement is crucial. We used the Job Demand-Resource (JD-R) model to test the association between neuroticism and work engagement. As neuroticism represents the stress-proneness of an individual, we further investigated if stress mediates the neuroticism-work engagement link. For the nurses, patient interaction is an integral part of their job. Based on the data collected from the nurses, we tested if contact with patients (i.e., beneficiary contact) alleviates the adverse effect of neuroticism on work engagement. During COVID-19, there was an intense need for nursing support. Hence, avoiding duty when society is looking for support might induce a fear of stigmatization among the nurses. We examined if the perceived stigma of duty avoidance would affect the neuroticism-engagement relationship. Our results indicated that higher patient contact alleviated the adverse effect of neuroticism on work engagement. On the other hand, higher fear of stigma exacerbated the adverse effect of neuroticism on work engagement. We further checked the combined effect of beneficiary contact and fear of stigma on neuroticism-work engagement relationships. The findings highlighted the importance of societal factors and policymakers in enhancing nurses’ work engagement.

## Introduction

Nurses play a critical role in providing quality care to COVID patients (Villar et al., 2021), preventing the collapse of healthcare systems. Apart from providing healthcare services, they helped in contact tracing, served quarantined clients in community care services, and worked toward preventing and handling the pandemic (Zhang, 2021). Their responsibilities included educating people on COVID-19 prevention and reducing misinformation about the virus (Choi et al., 2020). Nurses were expected to maintain a high standard of hygiene while providing support and care to patients (Hoogendoorn et al., 2021). Thus, nurses played a significant role in battling COVID-19 (Allobaney et al., 2022). However, long working hours in quarantine areas with inadequate and insufficient resources took a heavy toll on the nurses.

Further, due to changing policies and increased work hours, nurses face tremendous challenges personally and professionally (Wierenga and Moore, 2020). It was also due to an increased number of patients and higher absences among the health care workers due to sickness or quarantine protocols (Bernburg et al., 2021). Moreover, the fear of catching and passing the infection to family members created psychological challenges. These challenges increased nurses’ anxiety, depression, stress, and burnout. Scholars have highlighted the possibility that post-traumatic stress disorder among nurses (Mealer et al., 2009) might have harmful consequences in the long run. Thus, the pandemic exposed nurses to physical, psychological, and social challenges. Despite the odds, the nurses contributed to the society in battling the pandemic.

In the present study, we focused on work engagement for the following reasons. Work engagement is “a positive, fulfilling work-related state of mind that is characterized by vigor, dedication, and absorption” (Schaufeli et al., 2006, p. 702). For example, engaged employees achieve higher output and contribute toward team effectiveness (Turner, 2020). Engaged nurses are likely to inspire and keep up spirits in the wards, particularly during critical and low morale periods, improving patient care (Allisey et al., 2016). Further work engagement promotes employees’ psychological and physical health (Bakker and Demerouti, 2008). Scholars have attributed many factors that impact nurses ‘work engagement during the pandemic. For example, individual factors such as resilience, self-efficacy (Badu et al., 2020), feeling of belongingness, and societal factors such as support from the society (González-Sanguino et al., 2020) impact nurses’ work engagement. Improving our understanding of other factors that facilitate nurses’ work engagement during COVID-19 might help nurses, organizations, and policymakers to initiate necessary measures.

Since the nurses’ response during the pandemic was not uniform, this study aims to understand the role of personality concerning work engagement. In the present study, we focused on neuroticism, a personality dimension. Neuroticism is defined as “relatively stable individual differences in the tendency to experience negative affect (e.g., anxiety, sadness), to more readily perceive situations as threatening or stressful, and to respond quickly and strongly to such situations with greater negative affect” (Wrzus et al., 2021, p. 692). Highly neurotic individuals are likely to experience negative emotions (Eysenck, 1967), leading to extreme adverse reactions (Anicich et al., 2020). It makes individuals overly sensitive to threats, thereby susceptible to high stress (Gunthert et al., 1999), making it an essential predictor of individual health (Friedman et al., 2010). Neurotic individuals tend to convert ordinary situations into threatening situations (Widiger and Oltmanns, 2017) and react to stressful situations with distress (Swagler and Jome, 2005). This becomes a bigger problem in the COVID-19 scenario when the job of healthcare professionals is highly demanding. Nursing is a demanding occupation, and nurses experience intense emotions, such as stress and loneliness, in their workplace (Anand and Mishra, 2021).

Further, nurses would likely experience physical and mental health challenges during the COVID pandemic. The present study examined the direct and indirect effect (through perceived stress) of neuroticism on work engagement during COVID-19. Compared to an objective measure of stress, perceived stress captures the level of stress experienced by an individual; hence, it is a better predictor of employee outcomes (Cohen et al., 1983).

Moreover, patients are the beneficiaries of nurses’ work. The degree to which employees interact with the people who are touched by their work is termed beneficiary contact (Grant, 2007). It allows employees to witness the immediate effects of their work. Hence, we investigated if patient contact (termed beneficiary contact) ameliorates the adverse effect of neuroticism on work engagement. During the pandemic, healthcare employees, especially nurses, are expected to help humanity in whatever way possible. Hence, nurses abstaining from duty in this trying time are likely to get stigmatized by others. Fear of stigma denotes individuals’ perception of stigmatizing attitudes of others toward themselves (Berger et al., 2001). It involves distinguishing characteristics that devalue a person (Goffman, 1963). Therefore, it is an opposing force that might push the nurses to do their work. Hence, we further examined if the fear of stigmatization could impact neuroticism-work engagement linkage.

### Theory and hypotheses

We draw from the job demand-resource (JD-R) model to argue the linkage between neuroticism and work engagement. The JD-R framework classifies job characteristics into two broad categories: job demands and job resources (Bakker and Demerouti, 2007). Job demands are the “physical, psychological, social, or organizational aspects of the job that require sustained physical and/or psychological (cognitive and emotional) effort or skills and are therefore associated with certain physiological and/or psychological costs” (Bakker and Demerouti, 2007, p. 312). On the other hand, job resources promote motivation and, thus, “are functional in achieving work goals; reduce job demands and the associated physiological and psychological costs; and stimulate personal growth, learning and development” (Bakker and Demerouti, 2007, p. 312).

Neuroticism describes an individual as anxious, fearful, tense, nervous, defensive, and moody (Costa and McCrae, 1992). According to Eysenck (1967), the limbic system of neurotic individuals gets overwhelmed by stressful stimuli. As a result, neurotic individuals usually convert ordinary situations into threatening situations (Widiger and Oltmanns, 2017), as they are susceptible to anxiety-inducing environmental cues and possess a pessimistic worldview (Spector et al., 2000), leading to adverse perceptions of work situations. Scholars found a positive relationship between neuroticism and job demand (Bakker et al., 2010). During the pandemic, the nurses worked under intense job demands, such as a lack of critical care resources, ICU beds and PPE suits, and the absence of a definite cure (Nathan et al., 2020). Unlike others, neurotic nurses are likely to experience these work situations as more demanding. For example, neurotic individuals are likely to experience higher fear of COVID-19 (Caci et al., 2020). As j*ob demands* include physical, psychological, social, or organizational features of a job, it strains individuals. To cope with the strain, individuals distance themselves from stain-inducing work (Connor-Smith and Flachsbart, 2007). As work engagement represents vigor, dedication, and absorption to work, we propose that during COVID-19, neurotic nurses will display reduced work engagement.

*Hypothesis 1:* Neuroticism is negatively related to work engagement.

### Stress as a mediator of neuroticism and work engagement

According to the JD-R model, Job demands impose physiological and psychological costs (Bakker and Demerouti, 2007). COVID-19 has increased nurses’ workload due to an increased patient flow and the absence of colleagues owing to contracting COVID-19 and self-isolation due to close contact with an infected individual (Turale et al., 2020). Scholars suggest that neuroticism increases stress in two ways (Specter and Ferrari, 2000). First, neurotic individuals are more vulnerable to harmful stimuli. Scholars argue that neurotic individuals pay more attention to COVID-19-related information and are more concerned about the pandemic’s repercussions, showing higher stress levels (Liu et al., 2021). Thus, neurotic individuals respond to these events with negative emotions, further depleting their resources, and hence they indulge in ineffective behaviors (Van Jaarsveld et al., 2019). As a result, neuroticism is likely to enhance psychological strain, causing energy depletion (Penney et al., 2011).

Second, neurotic individuals tend to perceive ordinary situations as threatening. Neurotic individuals are more reactive to daily stressors (Suls et al., 1998) and react to stressful situations with intense distress (Swagler and Jome, 2005). It became a more significant problem during COVID-19 as the neurotic nurses perceived the job as highly demanding. Consequently, neuroticism is associated with negative views about self and others (Watson and Clark, 1984), leading to a wide range of adverse outcomes such as emotional exhaustion (Zellars et al., 2004) and health impairment (Bakker et al., 2010). In a highly stressful situation, individuals reduce unpleasant arousal by distancing themselves from stressors or related activities (Connor-Smith and Flachsbart, 2007), leading to disengagement (Bouchard, 2003). Hence, we posit that neurotic nurses experience enhanced stress leading to reduced work engagement.

*Hypothesis 2:* Perceived stress mediates the relationship between neuroticism and work engagement.

### Beneficiary contact

According to the JD-R model, job demands cost energy (Bakker, 2015), whereas job resources help individuals deal with these demands (Bakker and Demerouti, 2007). Moreover, the JD-R theory proposes that the interaction of job demands and resources shapes employees’ work and work outcomes. One critical job resource is contact with the beneficiaries.

Contact with beneficiaries increases task significance (Humphrey et al., 2007), interpersonal liking (Schoenrade et al., 1986), and employee motivation. Thus, beneficiary contact is a relational aspect of the job that provides employees with resources to strive for organizational goals (Vittal et al., 2022). When nurses have direct contact with the patients and can observe the effects of their work on health outcomes, they are likely to realize the criticality of their work. Extant research (Cohen and Rodgers, 2020) suggests that when the nurses were not allowed to remain in touch with their patients or could not see the changes in patient health during the pandemic, the quality of their care and engagement levels suffered. Nurses’ realization of the importance of their work is a positive resource. Thus, beneficiary contact is likely to buffer the adverse effect of neuroticism on employee engagement. On the other hand, neurotic nurses fail to realize the importance of their work and are likely to experience reduced work engagement. Based on the above discussion, we propose the following hypothesis.

*Hypothesis 3:* Beneficiary contact moderates the negative relationship between neuroticism and work engagement, such that the relationship is more negative when beneficiary contact is low.

### Fear of stigma

The social context of work influences individuals’ perceptions of the job they perform (Gergen, 2009). The pandemic made the nurses indispensable. The nurses contributed to society in an acute resource constraint environment. Due to the patient interface, they expose themselves to the virus infection leading to significant risk to their life and threat of contagion to family members. However, being a nurse and not contributing during the pandemic was devalued in society. A stigma is an “attribute or a characteristic that conveys a social identity that is devalued in a particular context, which includes being the target of negative stereotypes, being rejected socially, being discriminated against” (Crocker et al., 1998, p.505). Studies about the stigma against health care providers, such as the nurses during COVID-19, are minimal (Nashwan et al., 2022). Though there are many drivers of stigma, the absence of nurses from their job during the pandemic is a stigma worth exploring. As stigma represents social rejection, nurses are likely to experience the fear of stigma. It involves a distinguishing characteristic that induces the fear of being devalued in a social context (Goffman, 1963). Drawing from the JD-R model, Barbier et al. (2013) argued that stigmatization is a kind of demand like job demand. Hence, in the workplace, the fear of stigma is likely to increase job demand impacting individuals’ attachment, attitude to work (Shantz and Booth, 2014), and turnover intention (Pinel and Paulin, 2005).

Similarly, in a society, nurses are likely to experience the fear of stigma. The nurses are likely to experience more negative interpersonal treatments (Singletary and Hebl, 2009) due to avoiding their duty when there is an intense need. Major and O’Brien (2005) suggested that stigma elicits voluntary (e.g., coping efforts) and involuntary (e.g., anxiety, working memory load) responses threatening one’s identity. Neurotic individuals are likely to experience enhanced fear of stigma (Cyders and Smith, 2008). The fear of stigma may push the neurotic nurses to their workplace, but it is unlikely to engage them with their work due to perceived job demand and its adverse impact on mental and physical well-being (Barbier et al., 2013). Further, studies found that nurses who treat COVID-19 patients experience an adverse impact on their professional self-concept (Allobaney et al., 2022), which would have negatively impacted their work engagement and patient care (Randle and Arthur, 2007).

*Hypothesis 4:* The fear of stigma moderates the relationship between neuroticism and work engagement, such that the relationship is more negative when the fear of stigma is high.

**Figure 1:**
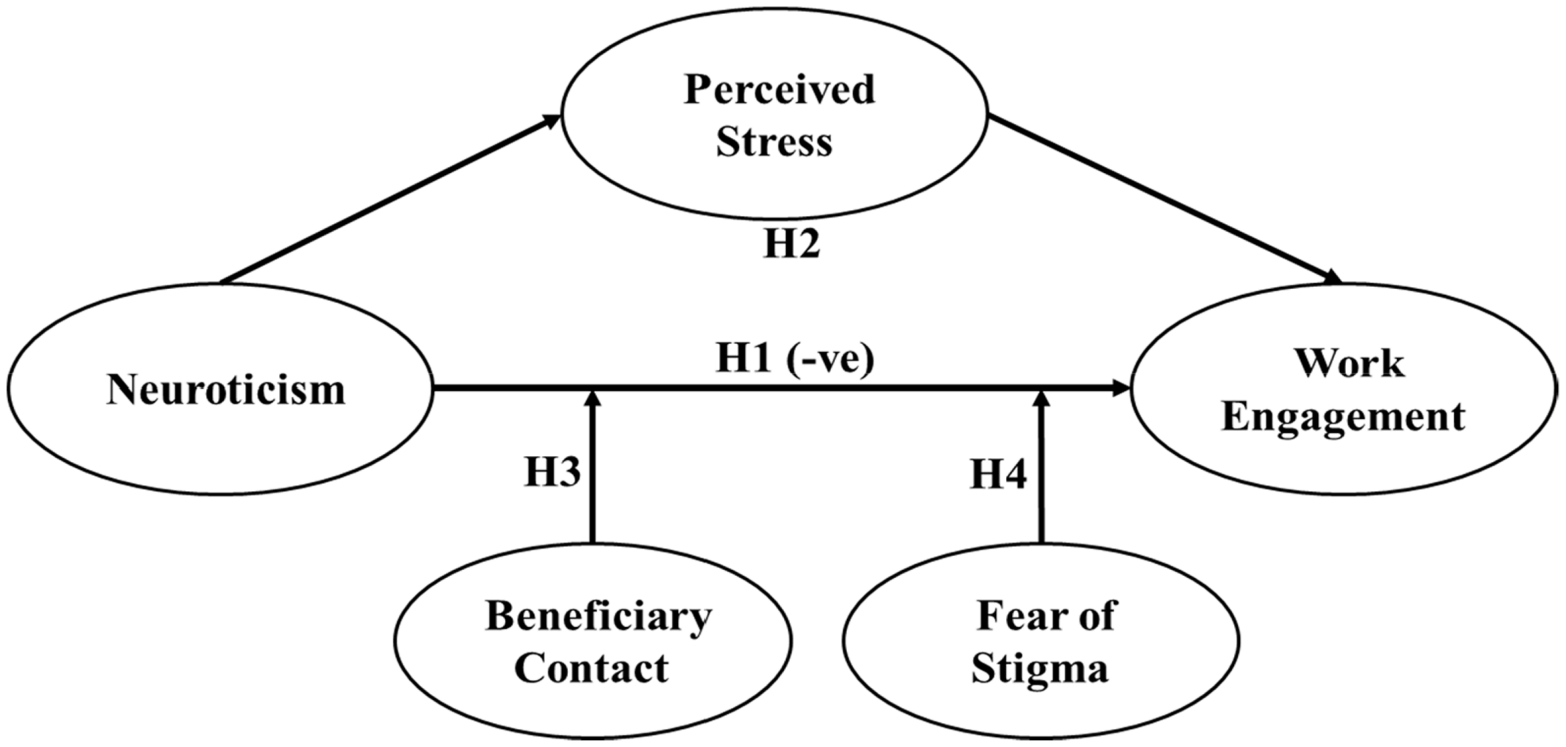
Hypothesized model. H1 indicates a negative relationship between neuroticism and work engagement; H2 indicates the indirect effect of neuroticism on work engagement through perceived stress. H3 and H4 indicate the moderation effect of beneficiary contact and fear of stigma, respectively, on the negative relationship between neuroticism and work engagement.

The hypothesized model is presented in Figure 1.

## Materials and Methods

### Participants

We collected the data after getting approval from the institutional review board. All the participants of the study provided oral consent before answering the questionnaire. We contacted 900 nurses, of which 790 agreed to participate in the study. Finally, we received 752 forms from the nurses. We contacted the nurses during their non-work time with prior appointments. Hence, there was no need to give reminders. After data cleaning, we included 657 usable responses from the nurses in the study. Of the 657 respondents, 445 were females and 212 were males. We denied the participation of some nurses in the survey as they had gone through some personal tragedy in the said period, so participating in the survey would have been emotionally exhausting for them. The required number of participants for performing linear regression using six predictors was observed to be 242 (effect size = 0.15, *α* = 0.01, power = 0.90) using GPower 3.1.9.4 (Faul et al., 2007).

We defined three explicit inclusion and exclusion criteria. First, we included only those nurses who had worked |in the COVID-19 ward during the second wave in India (March–June 2021). During this period, India reported at least 50,000 cases daily, with a peak of over 400,000 cases in May. We excluded the nurses from the study who did not serve in the COVID ward. Second, the nurses should be full-time practicing nurses and not student apprentices in their respective hospitals. Third, the hospitals should be in bigger cities, as bigger cities were the most affected in the country and faced a shortage of beds and medical facilities. We collected data from the nurses working in different hospitals in Delhi, Bangalore, Kolkata, Chennai, Ahmedabad, Mumbai, Pune, Hyderabad, and Lucknow.

In survey-based research, scholars have proposed multiple approaches to control the possibility of common method variance (Podsakoff et al., 2003). Maintaining a temporal separation while collecting the data is one of the powerful approaches. However, there is no specific accepted time interval between different phases of data collection (Cole and Maxwell, 2003). However, in recent scholarly work, researchers have maintained a temporal separation of 2 weeks to control the common method bias without losing the effect of the predictor on the outcome (Raja et al., 2018; Liekefett et al., 2022). Consistent with the existing practice, we followed a temporal separation of 2 weeks in our data collection. In phase 1, we captured age, gender, marital status, type of work, work experience, the city of work, neuroticism, and fear of stigma. In phase 2, we captured work experience, prosocial motivation, work engagement, beneficiary contact, and perceived stress. We used the respondent’s name, mobile number, and experience to match phase 1 data with phase 2. We collected the responses on a five-point scale ranging from 1 (Never/Strongly Disagree) to 5 (Always/Strongly Agree).

### Measures

#### Neuroticism

We used the eight-item scale of the Big-5 inventory to capture neuroticism (John and Srivastava, 1999). A sample item is “I am neither relaxed nor can handle stress well.” Studies have used this scale to measure neuroticism in the Indian context (Srivastava and Bajpai, 2020).

#### Beneficiary contact

We adapted four items from the measures developed by Grant (2012). A sample item reads, “My job involves a great deal of interaction with the patients.” Studies have used this scale in the Indian context (Vittal et al., 2022).

#### Fear of stigma

We wanted to measure the fear of the stigma associated with not going to work during COVID-19. Hence, we measured the fear of stigma by adapting the items of the occupational stigma scale (Schaubroeck et al., 2018). A sample item reads, “Most people would have disrespected me if I had not gone to work during the pandemic.”

#### Perceived stress

We measured perceived stress using the scale developed by Cohen et al. (1983). The items were adapted to fit into our context. A sample item is “In the last week, how often have you found that you could not cope with all the things that you had to do?.” The scale was used and validated in the Indian context (see Grover et al., 2020).

#### Work engagemen

We used the 9-item Utrecht Work Engagement Scale (version-9) to measure work **e**ngagement (Schaufeli et al., 2006). The scale has three dimensions, namely visor, dedication, and absorption. A sample item is “At my work, I feel bursting with energy.” Studies have used this scale in the Indian context (see Alok, 2013).

#### Control variables

We controlled for age, gender, marital status, type of work, work experience, city, and prosocial motivation as they are related to work engagement (Othman and Nasurdin, 2013; Goštautaitė and Bučiūnienė, 2015).

Before running the analyzes, we checked for convergent and discriminant validity. We conducted confirmatory factor analysis using structural equation modeling. The fit indices for a five-factor model were robust (CFI = 0.933, IFI = 0.933, TLI = 0.921, SRMR = 0.06, RMSEA = 0.040, & CMIN/df = 2) and better than the one factor, two factor, three factor and four factor models. For convergent validity, we relied on the Composite reliability (CR) scores following the recommendations by Fornell and Larcker (1981). Extant research (e.g., Lam, 2012) suggests that composite reliability scores higher than 0.70 is sufficient to establish convergent validity. The composite reliability for all the scales was greater than 0.80, establishing convergent validity. We further found that the AVE (average variance extracted) scores were greater than the square of the bivariate correlations, establishing discriminant validity (Fornell and Larcker, 1981). Apart from collecting the data at two time periods, we used an established marker variable (i.e., attitude toward blue color) to minimize common method variance (Miller and Simmering, 2022).

## Results

Table 1 presents the mean, standard deviation, bivariate correlations, and reliability scores of the study variables. We found a negative correlation between neuroticism and work engagement (*r* = −0.17, *p* < 0.001). Further, we found that neuroticism is positively related to perceived stress (*r* = 0.28, *p* < 0.001).

**Table 1:** Means, standard deviations, and zero-order correlations among the study variables.

Sl. No.	Variable	Mean	SD	1	2	3	4	5	6	7	8	9	10	11	12	13
1.	Age	28.53	5.13													
2.	Gender	1.68	0.47	−0.03												
3.	Marital Status	1.54	0.50	−0.55^***^	0.04											
4.	Organization Type	1.89	0.38	−0.36^***^	0.04	0.24^***^										
5.	Work Experience	5.23	3.81	0.73^***^	0.00	−0.46^***^	−0.29^***^									
6.	City	5.78	3.73	0.05	0.00	0.08^*^	−0.21^***^	0.03								
7.	PSM	4.27	0.62	−0.08^*^	−0.18^***^	0.04	0.14^***^	0.01	−0.12^**^	**0.74**						
8.	Marker Variable	3.36	1.30	−0.07	−0.07	0.08	0.12^**^	−0.14^***^	−0.05	0.03						
9.	Neuroticism	1.93	0.71	0.11**	0.24^***^	−0.09^*^	−0.12^**^	0.00	0.22^***^	−0.29^***^	−0.05	**0.84**				
10.	Fear of Stigma	3.41	1.09	−0.04	−0.22^***^	0.10^**^	−0.03	0.00	0.05	0.10^**^	0.19^***^	−0.44^***^	**0.88**			
11.	Beneficiary Contact	3.60	0.89	0.05	0.05	−0.05	−0.13^**^	0.10*	0.10^**^	0.03	−0.13^**^	0.06	−0.13^**^	**0.75**		
12.	Perceived Stress	2.45	0.59	0.04	0.09^*^	0.01	−0.16^***^	0.05	0.39^***^	−0.14^***^	−0.18^***^	0.28^***^	−0.22^***^	0.12^**^	**0.73**	
13.	Work Engagement	4.35	0.45	−0.13^**^	−0.04	0.08^*^	0.13^**^	−0.06	0.12^**^	0.14^***^	0.05	−0.17^***^	0.09^*^	−0.01	−0.19^***^	**0.79**

*N* = 657; Standardized correlation analysis were reported, ^***^p < 0.001; ^**^p < 0.01; ^*^p < 0.05. SD, Standard deviation; PSM, Prosocial motivation; City, City from which data were collected. ^***^p < 0.001, ^**^p < 0.01; ^*^p < 0.05. The values in the diagonal (in bold) represent the Cronbach scores of the corresponding variables.

We eliminated the effect of control variables and the marker variable while regressing work engagement with neuroticism (see Table 2). We found a significant negative relationship (*β* = 0.16, *p* < 0.001, Δ*R*^2^ = 0.02). Hence, hypothesis 1 was supported.

**Table 2:** Moderation effect of fear of stigma and beneficiary contact on neuroticism-work engagement relationship.

Predictor	Step 1 *β* (SE)	Step 2 *β* (SE)	Step 3A *β* (SE)	Step 3B *β* (SE)	Step 4A *β* (SE)	Step 4B *β* (SE)	Step 5 *β* (SE)	Step 6 *β* (SE)	*R* ^2^	Δ*R*^2^
Control variable										
Age	−0.14^*^ (0.01)	−0.12^*^ (0.01)	−12^*^ (0.01)	−0.12^*^ (0.01)	−0.10 (0.03)	−0.11 (0.01)	−0.10 (0.01)	−0.10 (0.01)	**0.07**	**0.07**
Gender	−0.02 (0.03)	0.01 (0.04)	0.01 (0.04)	0.01 (0.04)	0.02 (0.04)	0.02 (0.04)	0.02 (0.04)	0.02 (0.04)		
Marital status	−0.01 (0.04)	−0.03 (0.04)	−0.03 (0.04)	−0.03 (0.04)	−0.02 (0.04)	−0.02 (0.04)	−0.02 (0.04)	−0.02 (0.04)		
Organization type	0.12^**^ (0.05)	0.12^**^ (0.05)	0.11^**^ (0.05)	0.11^**^ (0.05)	0.10^*^ (0.05)	0.12^**^ (0.05)	0.11^**^ (0.05)	0.11^*^ (0.05)		
Work experience	0.07 (0.00)	0.05 (0.01)	0.05 (0.01)	0.05 (0.01)	0.04 (0.01)	0.04 (0.01)	0.03 (0.01)	0.04 (0.01)		
City	0.16^***^ (0.01)	0.19^***^ (0.01)	0.20^***^ (0.01)	0.19^***^ (0.01)	0.20^***^ (0.01)	0.20^***^ (0.01)	0.21^***^ (0.01)	0.20^***^ (0.01)		
PSM	0.12^**^ (0.03)	0.09^*^ (0.03)	0.09^*^ (0.03)	0.09^*^ (0.03)	0.09^*^ (0.03)	0.09^*^ (0.03)	0.09^*^ (0.03)	0.09^*^ (0.03)		
Marker variable	0.04 (0.01)	0.04 (0.01)	0.04 (0.01)	0.04 (0.01)	0.04 (0.01)	0.04 (0.01)	0.04 (0.01)	0.04 (0.01)		
Independent variable										
Neuroticism		−0.16^***^ (0.03)	−0.16^***^ (0.03)	−0.16^***^ (0.03)	0.16 (0.10)	−0.56^**^ (0.11)	−0.22 (0.17)	−1.67^*^ (0.47)	**0.09**	**0.02**
Moderating variable										
Beneficiary contact				0.00 (0.02)		−0.27^*^ (0.06)	−0.20 (0.12)	1.20^*^ (0.27)	**0.09**	**0.00**
Fear of Stigma			0.00 (0.02)		0.26 (0.06)		0.32 (0.10)	−0.90 (0.26)	**0.09**	**0.00**
Interaction term										
Neuroticism × Beneficiary Contact						0.50^*^ (0.03)	0.47^†^ (0.03)	2.23^*^ (0.12)	**0.10**	**0.01**
Neuroticism × Fear of Stigma					−0.34^*^ (0.03)		−0.34^*^ (0.03)	1.18 (0.12)	**0.10**	**0.01**
Beneficiary Contact × Fear of Stigma							−0.08 (0.02)	1.35^†^ (0.07)	**0.10**	**0.00**
Neuroticism × Beneficiary Contact × Fear of Stigma								−1.77^*^ (0.03)	**0.11**	**0.01**

*N* = 657; Standardized beta values (*β*) are reported; SE, standard error; PSM, Prosocial motivation; City, City from which data were collected; ^†^*p* < 0.1; ^*^p < 0.05; ^**^p < 0.01; ^***^p < 0.001.

We tested for the mediation effect of perceived stress on the relationship between neuroticism and work engagement using Model-4 of PROCESS Macro (Preacher and Hayes, 2008). The indirect effect of neuroticism on work engagement was statistically significant based on 5,000 bootstrap samples (see Table 3). The bootstrapping at 95% confidence intervals does not include zero (−0.04, −0.02). We further conducted structural equation modeling to test the model fit of the mediation effect. The fit indices indicate the robustness of the model (GFI = 0.926, IFI = 0.939, TLI = 0.924, SRMR = 0.06, RMSEA = 0.043, & CMIN/df = 2.2). Hence hypothesis 2 was supported.

**Table 3:** Mediation effect of perceived stress on neuroticism-work engagement relationship.

	Model 1 *β* (SE) (perceived stress)	Model 2 *β* (SE) (work engagement)
Neuroticism	0.14 (0.03)	−0.08^**^(0.03)
Perceived stress		−0.03^***^
*R* ^2^	0.24	0.14
*F*	20.7	9.92
95% CI bootstrap results (lower and upper)		(−0.04, −0.02)

*N* = 657; Standardized beta values are reported; SE, standard error.^*^p < 0.05; ^**^p < 0.01; ^***^p < 0.001.

**Figure 2:**
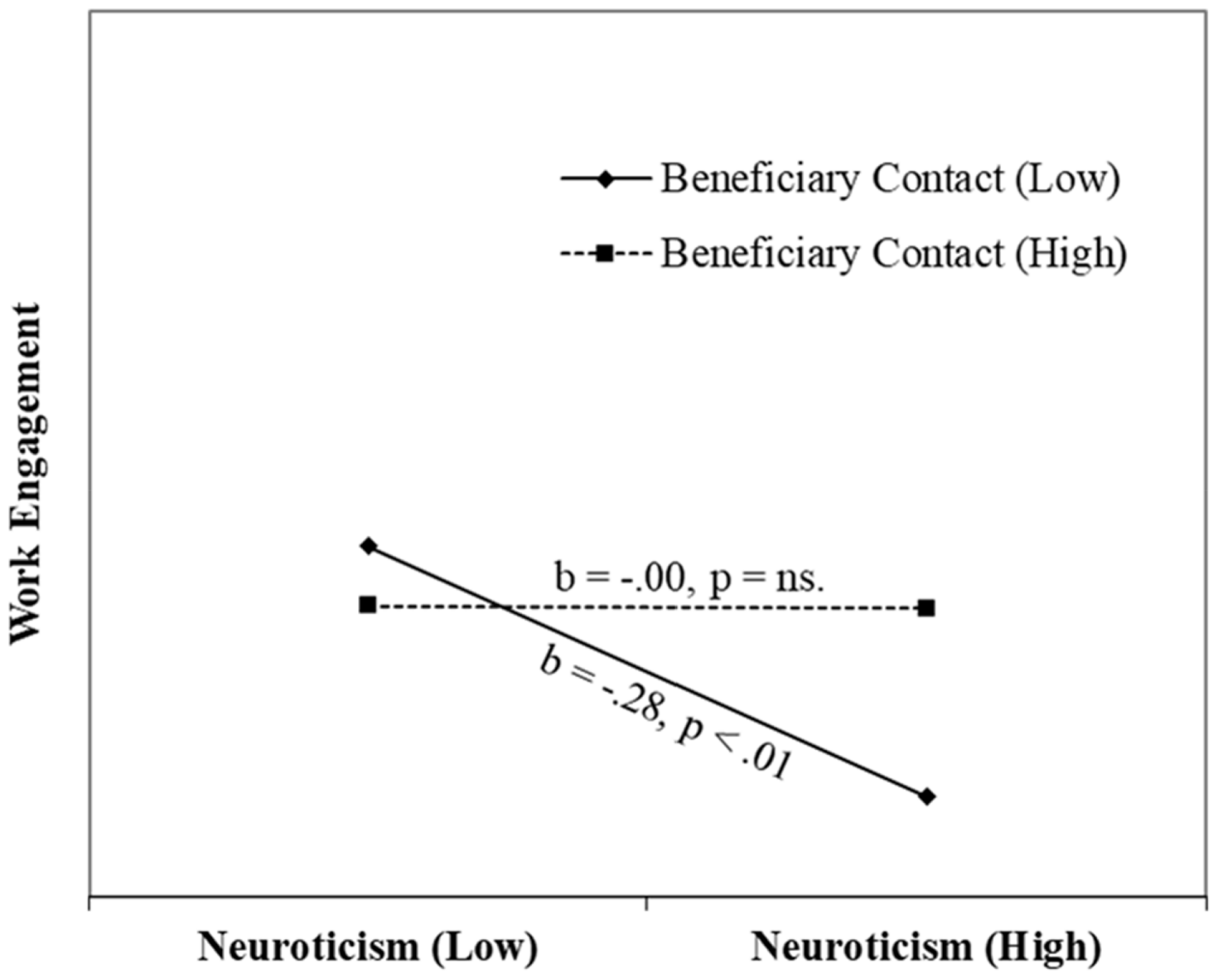
Interaction effect of beneficiary contact on Neuroticism–Work Engagement relationship.

We conducted a regression analysis to test the moderation effect of beneficiary contact on the neuroticism-work engagement relationship. Our results (see Table 2) indicated a significant positive effect of the interaction term (neuroticism*beneficiary contact) on work engagement (*β* = 0.50, *p* < 0.05, Δ*R*^2^ = 0.01). The interaction effect explained an additional 1 % variance after controlling the effect of control variables, marker variable, independent variable, and moderator. We plotted the relationship between neuroticism and work engagement for high and low values of beneficiary contact (see the graph in Figure 2).

**Figure 3:**
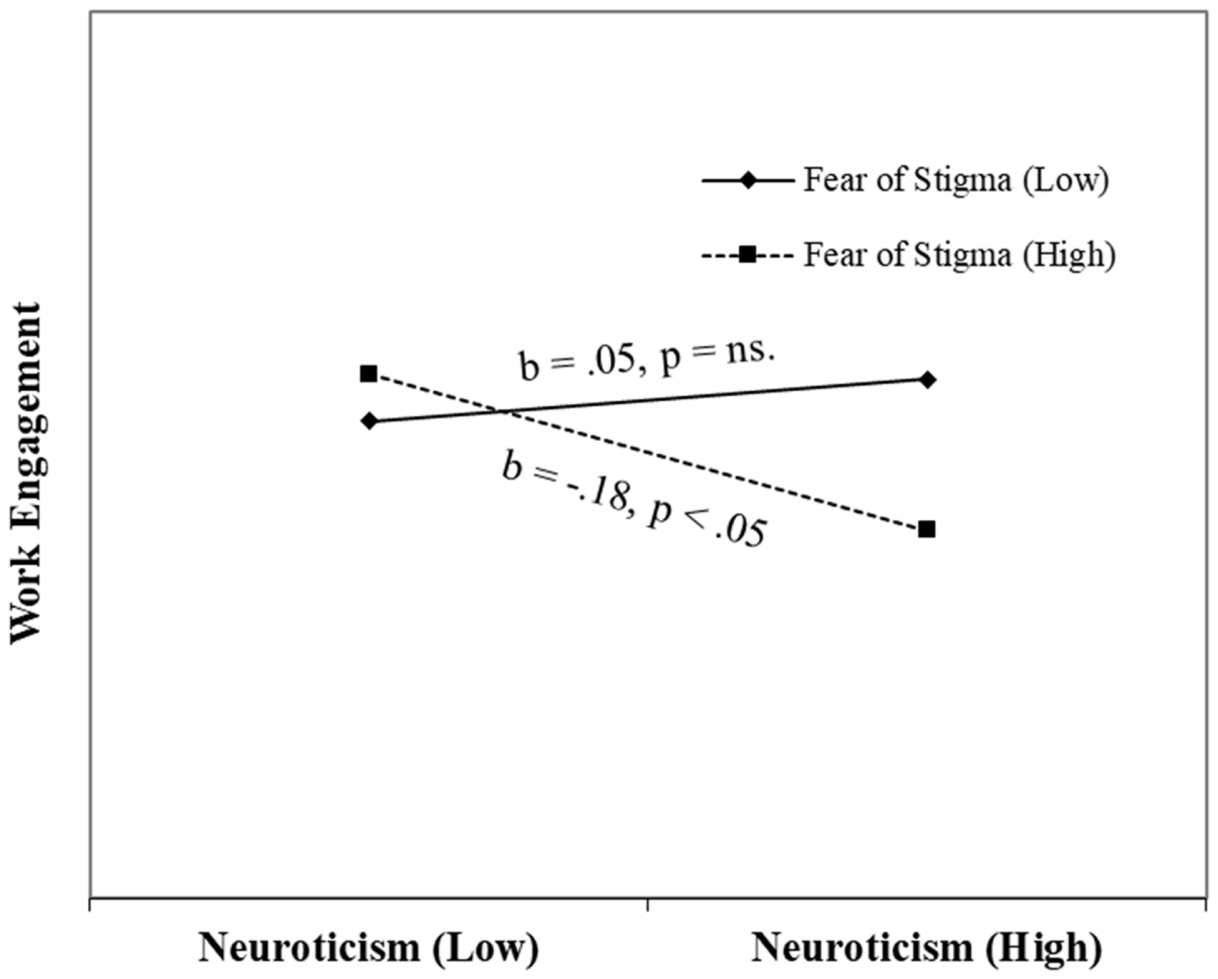
Interaction effect of perceived stigma on Neuroticism–Work Engagement relationship.

The graph indicates the slope of the regression line is more negative when beneficiary contact is low. Thus, hypothesis 3 was supported. We repeated the above analysis to test the moderation effect of fear of stigma on the above relationship. As shown in Table 2, our results indicated that the fear of stigma has a significant moderation effect (*β* = −0.34, *p* < 0.05, Δ*R*^2^ = 0.01) on the relationship between neuroticism and work engagement. We plotted the relationship between neuroticism and work engagement for high and low values of perceived stigma (see the graph in Figure 3).

The graph indicates the slope of the regression line is more negative when fear of stigma is high. Hence, hypothesis 4 was supported.

### Additional analysis

**Figure 4:**
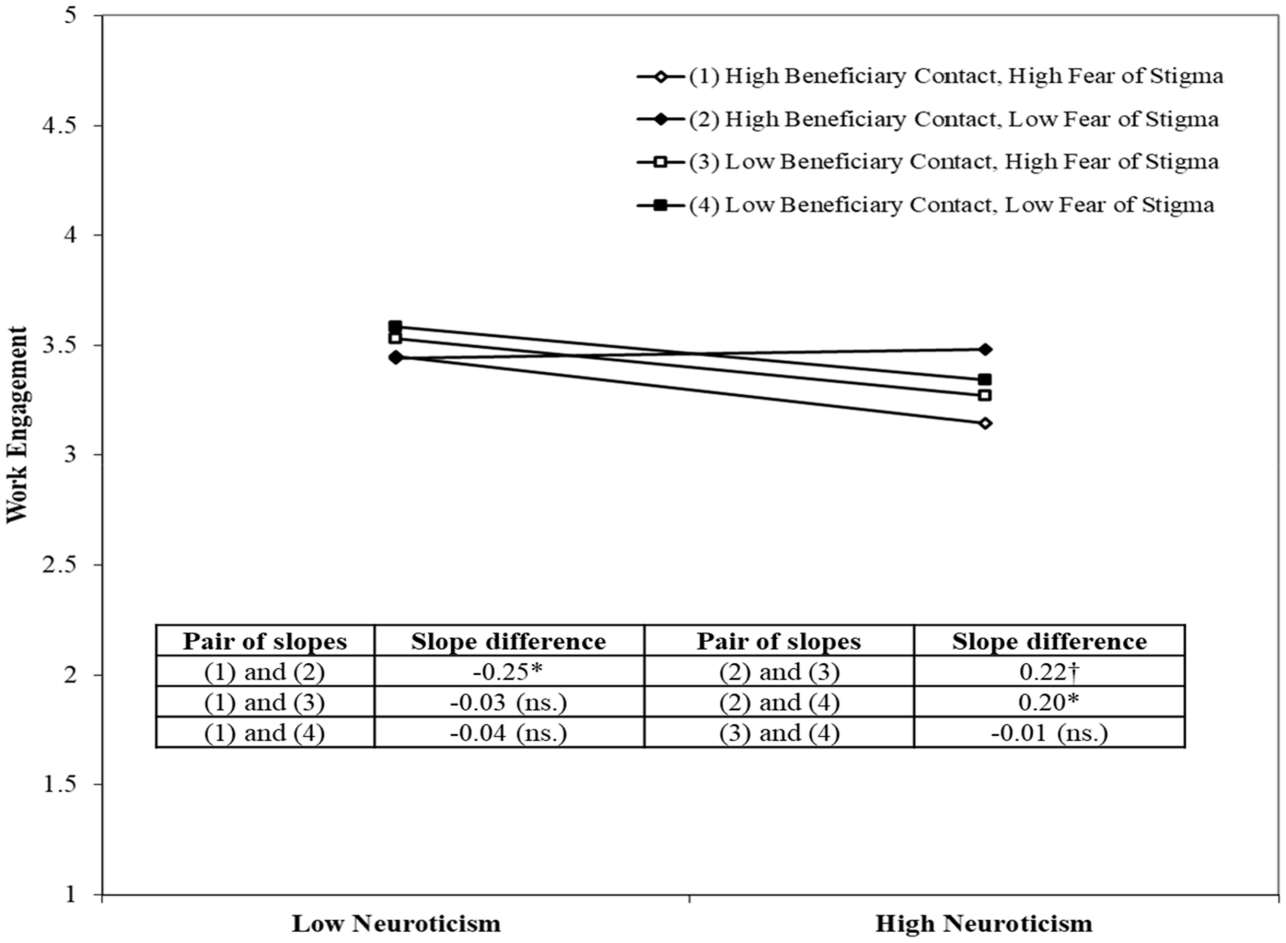
Interaction effect of beneficiary contact and fear of stigma on Neuroticism–Work Engagement relationship.

We conducted three additional analyzes to test the robustness of our findings. One, we conducted the analyzes without the control variables. We found consistent results. Second, we conducted the moderated mediation analysis, and the findings support our hypotheses. Third, we conducted the effect of both moderators on the relationship between neuroticism and work engagement. The analysis is provided in Table 3 (see Steps 5 and 6). We found that the interaction term (i.e., the interaction of neuroticism, beneficiary contact, and the fear of stigma) is significantly related to work engagement (*p* < 0.05; ΔR^2^ = 0.01) after controlling the effect of other relevant variables. We further presented the 3-way interaction graph in Figure 4.

We analyzed the linkage between neuroticism and work engagement by comparing responses having high beneficiary contact (and low fear of stigma) with low beneficiary contact (and high fear of stigma). We found the neuroticism-engagement linkage is more negative for low beneficiary contact (with high fear of stigma) compared to the reverse scenario (Slope difference: 0.22, *p* < 0.1). The finding is consistent with our arguments for hypotheses 3 and 4. We further compared the above linkage for respondents with low fear of stigma (and having high and low beneficiary contact). The findings indicate that in situations of low fear of stigma, the high beneficiary contact nullifies the adverse consequence of neuroticism on engagement (slope difference: 0.20, *p* < 0.05). The finding supports hypothesis 3. However, in situations of high fear of stigma, the negative effect of neuroticism on engagement does not get impacted by high or low beneficiary contact (slope difference: −0.03, *p* = ns.).

We further checked the impact of fear of stigma on the neuroticism-engagement relationship for respondents having high beneficiary contact. Our findings indicate that in situations of high beneficiary contact, the neuroticism-engagement linkage becomes more negative for high fear of stigma (slope change: −0.25, *p* < 0.05). However, in situations of low beneficiary contact, the fear of stigma (irrespective of high or low) does not impact the neuroticism-engagement linkage (slope difference: −0.01, *p* = ns.). The findings provide an additional layer of interpretation to hypothesis 4 by providing the situation when fear of stigma becomes critical. Finally, we checked the linkage between neuroticism and engagement when both fear of stigma and beneficiary contact are high compared to the situation when both are low. We found the slope difference is insignificant (slope difference: −0.04, *p* = ns.). The findings support complementarity, i.e., the adverse effect of high fear of stigma is complemented by high beneficiary contact.

## Discussion

The health crisis due to the pandemic poses threats to individual lives and society at large. Scholars have argued that along with contextual conditions, individual dispositions such as neuroticism could be a source of adverse outcomes during the pandemic (Caci et al., 2020). Our study brings two contradictory aspects to explain the work engagement of nurses. On the one hand, nurses as trained to help patients recover from medical ailments. Hence, nurses are likely to come forward to contribute to healthcare services during the pandemic.

On the other hand, neurotic individuals experience negative affect (Kroencke et al., 2020), fear responses (Sep et al., 2019), and undergo stressful life experiences (Penley and Tomaka, 2002). As a result, they are likely to disengage from stressful situations, such as attending the COVID-19 patients. Hence, it is crucial to understand the approach of neurotic nurses to their work during the pandemic. Though studies explain the effect of neuroticism on individual outcomes, such as stress, loneliness, and boredom during the pandemic (Caci et al., 2020; Ikizer et al., 2022), we focused on organizational outcomes such as work engagement. Nurses’ work engagement has societal implications and was critical during the pandemic. We found that neurotic nurses display reduced engagement in their work directly and indirectly through increased stress perception. Our results indicate a negative association between stress and work engagement among Indian nurses, which is in line with the research undertaken in other countries like Germany (Bernburg et al., 2021), China (Zhang et al., 2021), and Spain (Allande-Cussó et al., 2021). These findings highlight the importance of keeping stress low among employees to increase their work engagement, irrespective of the context or culture.

Further, we selected beneficiary contact and the fear of stigma (for not attending the job) as moderators of the above relationship. We selected the above two factors for the following reason. Beneficiary contact might pull (motivate) the nurses toward the job, whereas the fear of stigma for not attending the job might push (force) them to work but disengage from work itself. We found beneficiary contact reduces the negative linkage between neuroticism and work engagement. Further, the fear of stigma increases the negative relationship between neuroticism and work engagement. The results reveal that neurotic people are more likely to disengage from work due to their personality traits, i.e., the tendency to focus on negative aspects during the pandemic. In the additional analysis we further tested the relative importance of push (fear of stigma) and pull (beneficiary contact) factors in explaining the neuroticism-work engagement relationship. Our findings highlighted the importance of both the factors. Firstly, we found that in cases of low fear stigma, high beneficiary contact nullifies the negative effect of personality trait of neuroticism on work engagement, and thus increases work engagement. Studies so far (e.g., Cernasev et al., 2021) have highlighted the importance of organizational policies and peer support in reducing stigma (push factor) to improve patient care. Our study highlights the additional advantage of beneficiary contact (pull factor) even in absence of push factors. Secondly, we found the negative effect of low beneficiary contact was offset by low fear of stigma and vice versa in the neuroticism-work engagement relationship. Thus, our study provides support in favor of complementarity of both push and pull factors. This is consistent with extant research highlighting that push and pull factors co-exist among healthcare employees (Mano-Negrin and Kirschenbaum, 1999).

### Contribution to theory

Studies in psychology have examined the adverse impact of neuroticism during the pandemic. However, studies on neuroticism in nurses are sparse. We examined the effect of neuroticism on their work engagement during the pandemic. Extant research (Hashish and Ashour, 2020) emphasized the importance of the push-pull factors in studies relating to nurses. Our study highlights the individual and combined influence of push (fear of stigma) and pull (beneficiary contact) factors on the relationship of neuroticism with work engagement during the pandemic. The stigma literature is well developed both within and outside the organization. However, the interplay of outside stigma on employee behavior is relatively unaddressed. We extended the stigma literature by constructing a new concept, fear of stigma. We focused on the stigma associated with not going to work rather than the stigma associated with work. Our study highlighted that the fear of stigma is a more potent force pushing nurses toward their work.

Our study demonstrated both the pull and push factors and their impact on nurses’ work and work. The pull factor (beneficiary contact) has a positive effect: it minimizes the adverse effect of neuroticism on work engagement. The push factor (fear of stigma) has an adverse effect. The fear pushes the nurses to do their work but reduces their work engagement. We further analyzed the interaction of push and pull factors to explain the neuroticism-work engagement relationship. We found that in situations of low fear of stigma, the high beneficiary contact nullifies the adverse consequence of neuroticism on engagement. But the positive effect of beneficiary contact does not hold when the fear of stigma is high.

Further, we found that in situations of high beneficiary contact, the neuroticism-engagement linkage becomes more negative for high fear of stigma. But in situations of low beneficiary contact, the fear of stigma loses its effect. Our study thus provides evidence of the complementarity of beneficiary contact (pull factor) and the fear of stigma (push factor). Our study further highlights the importance of fear of stigma in patient interface occupations. Traditionally the literature has focused on the factors within the organization. To understand their interplay with neuroticism in predicting work engagement, we have considered two factors outside the organization: positive (beneficiary contact) and negative (the fear of stigma). Though scholars have highlighted the social aspects of the job (Grant, 2012), the literature has predominantly focused on the positive aspects of the job. Our findings extend the job characteristics model by highlighting the job’s positive and negative social aspects.

### Contribution to practice

Our study proposes several inputs to the practitioners and policymakers. One, the study indicates that mere contact with beneficiaries mitigates the negative effect of neuroticism on work engagement. Organizations may find out suitable mechanisms to facilitate beneficiary contact with their nurses. Our findings indicated that pull factors are better than push factors in reducing the adverse effect of personality factors on work engagement. Organizations may find ways to motivate nurses rather than induce fear for their work engagement. Our recommendation to organizations is to focus on improving working conditions, and professional situations as these would improve the quality of nurses’ lives and their work engagement (Giménez-Espert et al., 2020).

Our study found that factors beyond the organization, i.e., societal factors, can influence employee engagement. Hence, policymakers can play a significant role in enhancing employee engagement. Communicating to people about the contributions of nurses and recognizing their efforts would transform the fear of stigma (push factor) into a call of duty (pull factor). Our study thus raises an important question. Why the responsibility for employee engagement lies within the organization? Policymakers may communicate many factors related to nurses’ work, such as the challenging work conditions (e.g., acute resource constraints), the threat of contamination, and long working hours, especially during critical times. It might bring dignity to their work (e.g., nursing is considered dirty work). The policymakers may find ways to pull the organizations for a societal cause rather than push them to deliver.

### Limitations and directions for future research

Our study has limitations. We conducted the study on a sample of Indian nurses. Unlike India, nurses in developed countries enjoy better pay and higher status. Hence, the findings may be helpful in countries having similar economic and social backgrounds. The literature supports that organizational systems and processes drive employee engagement. Future studies may explore if reward and recognition minimize the negative effect of neuroticism on work engagement. Studies have argued that in addition to beneficial contact, prosocial impact and worth of work influence frontline employees (Grant, 2012). Future studies might extend our study to examine the interplay of prosocial motivation with neuroticism in explaining work engagement in critical situations. Our findings indicate that females are more prone to the fear of stigma. Future studies may examine our model for the gender groups. We collected the data in two time periods during the pandemic. Though we have a solid theoretical underpinning, given the study’s cross-sectional nature, unequivocal causality cannot be established.

## Conclusion

The present study is one of the initial studies that explained neuroticism’s direct and indirect effects on nurses’ work engagement. We identified two critical factors (pull and push) that influenced the neuroticism-work engagement relationship. Our work highlighted an essential yet neglected issue: the role of societal factors on employee engagement. Our study demonstrated the criticality of social aspects of work on nurses’ work engagement. By demonstrating the criticality of organizational actions (fostering beneficiary contact) and the broader social fabric (minimizing the fear of stigma), our study brings the nurses to the center of the discussion.

## Data availability statement

The raw data supporting the conclusions of this article will be made available by the authors, without undue reservation.

## Ethics statement

The studies involving human participants were reviewed and approved by Institutional Review Board, IIM Indore. Written informed consent for participation was not required for this study in accordance with the national legislation and the institutional requirements.

## Author contributions

MV, SM, and HR: conceptualization, methodology, validation, software, formal analysis, writing—original draft, and writing—review and editing. AP: review and editing. All authors contributed to the article and approved the submitted version.

## Conflict of interest

The authors declare that this study received funding from GIZ, GmbH, India. The funder was not involved in the study design, collection, analysis, interpretation of data, the writing of this article or the decision to submit it for publication.

## Publisher’s note

All claims expressed in this article are solely those of the authors and do not necessarily represent those of their affiliated organizations, or those of the publisher, the editors and the reviewers. Any product that may be evaluated in this article, or claim that may be made by its manufacturer, is not guaranteed or endorsed by the publisher.
